# Differences in Expression of Human Leukocyte Antigen Class II Subtypes and T Cell Subsets in Behçet’s Disease with Arthritis

**DOI:** 10.3390/ijms20205044

**Published:** 2019-10-11

**Authors:** S. M. Shamsul Islam, Hyoun-Ah Kim, Bunsoon Choi, Ju-Yang Jung, Sung-Min Lee, Chang-Hee Suh, Seonghyang Sohn

**Affiliations:** 1 Department of Biomedical Science, Ajou University School of Medicine, Suwon 16499, Korea; shamsulislam21@gmail.com; 2 Department of Rheumatology, Ajou University School of Medicine, Suwon 16499, Korea; nakhada@naver.com (H.-A.K.); serinne20@hanmail.net (J.-Y.J.); dsm0217@naver.com (S.-M.L.); 3 Department of Microbiology, Ajou University School of Medicine, Suwon 16499, Korea; blueppang@aumc.ac.kr

**Keywords:** Behçet’s disease, inflammation, arthritis, HLA-DP, HLA-DQ, HLA-DR

## Abstract

It has been reported Human Leukocyte Antigen (HLA) gene polymorphism is a risk factor for the development of Behçet’s disease (BD). In this study, the association of HLA class II subtypes HLA-DP, DQ, DR, and T cell subsets in BD patients with arthritis was evaluated. Frequencies of HLA-DP, DQ, DR positive cells, and T cell subsets in peripheral blood leukocytes (PBL) were measured by flow cytometric analysis in BD, and compared to rheumatoid arthritis as disease controls and healthy controls. Frequencies of HLA-DQ were significantly decreased in whole PBL and granulocytes of BD active patients as compared to healthy controls. In monocytes populations, proportions of HLA-DR positive cells were significantly increased in BD active patients as compared to healthy controls. Proportions of CD4+CCR7+ and CD8+CCR7+ cells were significantly higher in BD active patients than in BD inactive in whole PBL. Frequencies of CD4+CD62L- and CD8+CD62L- cells in lymphocytes were significantly decreased in active BD than those in inactive BD. There were also correlations between disease activity markers and T cell subsets. Our results revealed HLA-DP, DQ, and DR expressing cell frequencies and several T cell subsets were significantly correlated with BD arthritis symptoms.

## 1. Introduction

Behçet’s disease (BD) is an inflammatory multisystemic vasculitis characterized primarily by recurrent mucosal, genital, and ocular inflammation. Although its etiology remains obscure, it is believed that BD is triggered by viral, environmental, and genetic factors. Pro-inflammatory innate immune system derived activation or autoantigen is also considered as a causing factor in BD [1,2,3]. It has also been reported human leukocyte antigen (HLA) haplotypes play an important role in BD [4,5]. HLA class II molecules are cell surface glycoproteins, expressed on antigen presenting cells (APCs) such as macrophages, dendritic cells, endothelial cells, and other organ-specific APCs [6]. HLA class II molecules play an essential role in the adaptive immune system by displaying antigen peptides on CD4+ T cells. Many HLA-associated diseases have autoimmune features. An autoimmune process is inherently dependent on T-cells [7]. T cell-dependent immune responses are also regulated by HLA class II subtypes DP, DQ, and DR. Aberrant expression of HLA class II could be important in autoimmunity [8]. DR11 and DQB1*0301 are more frequent in HLA-B51 positive BD patients. DQ5 is negatively associated with BD, especially in HLA-B51 positive BD patients [9].

T cells play an important pathogenic role in BD [10]. However, it remains elusive concerning the mechanism of these pathogenic T cells responses. T cells carry T cell receptor (TCR) which encounters with antigens presented by APCs. Naïve T cell proliferation and differentiation are essential for optimal host defense [11]. Tissue homing effector and lymph node homing non-effector memory T cells can be distinguished by the expression of CCR7 [3]. CCR7 and CD62L are essential for lymphocyte migration [12,13]. CCR7 positive memory cells are named as central memory cells due to their potential of homing to secondary lymphoid tissue while CCR7 negative memory cells are named as effector memory cells due to their rapid effector function in peripheral lymphoid tissues [14]. Differentiation of T cell subsets also plays a pathogenic role in BD [15]. 

In this study, we provided the first evidence of the association of HLA-DP, DQ, DR, and differentiated T cells including naïve, effector, and memory T cells in peripheral blood leukocytes (PBL) of BD patients with arthritis. The correlation between the frequencies of these markers and clinical status were compared. Cell frequencies with these protein markers were quantified in peripheral blood leukocytes from BD patients with arthritis using flow cytometry (FACS). 

## 2. Results

### 2.1. Clinical Characteristics of Patients

Clinical characteristics of patients with BD are summarized in Table 1. Blood sampling was performed twice (1st at the active stage and 2nd at the inactive stage of the disease). The second sampling was performed after improvement in joint symptoms. Disease severity scores were significantly decreased at the 2nd sampling compared to that at the 1st sampling (1.27 ± 1.0 *vs.* 3.04 ± 1.0, *P* = 0.007). 

Erythrocyte sedimentation rate (ESR) was also decreased at the 2nd sampling, but was not significant (18.3 ± 13.6 mm/h *vs.* 29.8 ± 25.8 mm/h, *P* = 0.083). Medication is shown in Table 2. The use of colchicine in 19 (76.2%) patients, glucocorticoid in 21 (84.0%) patients, azathioprine in 7 (28.0%) patients, bucillamine in 1 (8%) patient, hydroxychloroquine in 8 (32%) patients, sulfasalazine in 10 (40.0%) patients, and nonsteroidal anti-inflammatory drugs in 16 (64.0%) patients was found. 

### 2.2. Frequencies of HLA-DP, DQ, and DR Positive Cells in Active BD Patients

**Figure 1:**
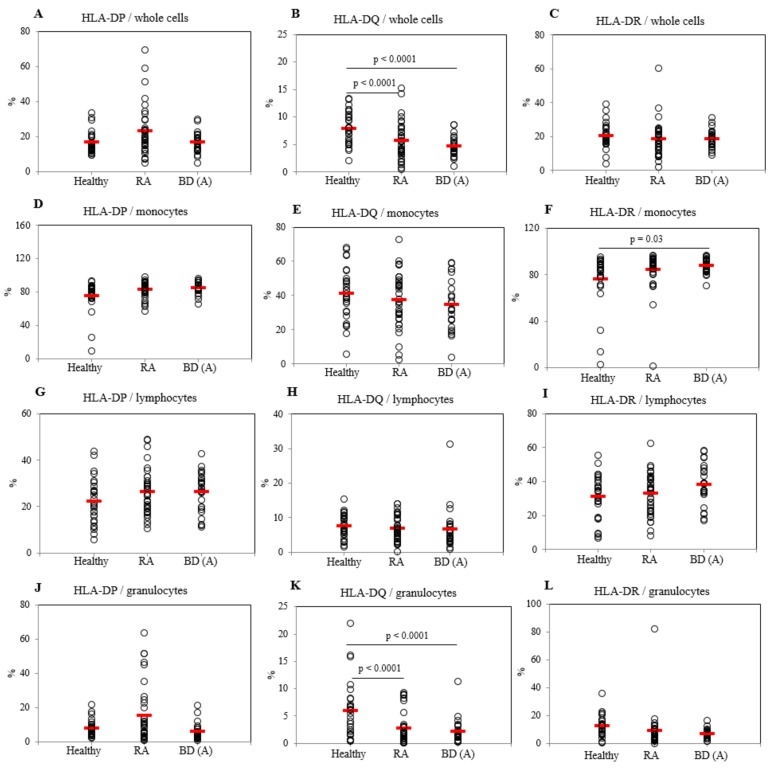
Frequencies of HLA-DP (**A**), DQ (**B**), and DR (**C**) in whole cells, monocyte (**D**–**F**), lymphocytes (**G**–**I**), and granulocytes (**J**–**L**) of healthy controls (HC), rheumatoid arthritis (RA), and active Behçet’s disease (BDA) patients. Isolated peripheral blood leukocytes (PBL) were subjected to flow cytometric surface staining. Results were obtained from 28 HC, 36 patients with RA, and 25 patients with BDA. The horizontal line specifies the mean value for each group.

The frequencies of HLA-DP, DQ, and DR positive cells were analyzed by flow cytometry. Frequencies of HLA-DQ in PBL whole cells were significantly decreased in active BD (BDA) patients (4.65 ± 1.80%, *p* < 0.0001) and in rheumatoid arthritis (RA) patients (5.66 ± 3.44%, *p* < 0.0001) compared to those in healthy control (HC) (7.92 ± 2.99) (Figure 1B). Frequencies of HLA-DP and HLA-DR expressing cells in PBL whole cells of BDA and RA patients showed no significant difference compared to those with HC (Figure 1A,C). HLA-DR expressing cell frequencies in monocytes of BDA patients were higher (87.49 ± 6.05%, *p* = 0.03) than those of HC (76.31 ± 22.94%) (Figure 1F). HLA-DP and HLA-DQ positive cell populations in monocytes of BDA patients were not significantly different from those of HC (Figure 1D,E). There were no significant differences observed in the frequencies of HLA-DP, DQ, and DR expressing cells in lymphocytes between BDA and HC groups (Figure 1G–I). Whereas, HLA-DQ expressing cell frequencies in granulocytes of BDA patients (2.14 ± 2.77%, *p* < 0.0001) and in RA patients (2.73 ± 2.96%, *p* < 0.0001) were significantly decreased compared to those of HC (5.87 ± 5.27%) (Figure 1K). Frequencies of HLA-DP and HLA-DR in granulocytes of BDA and RA patients were not significantly different compared to those of HC (Figure 1J,L). Proportion of HLA-DQ (*p* = 0.09) and HLA-DR (*p* = 0.05) in granulocytes were different between active and inactive BD by Wilcoxon-rank test analysis (Supplementary Table S1). Representative histograms of HLA-DP, DQ, and DR expressing cells in whole cells, lymphocytes, and granulocytes are shown in Supplementary Figure S4A–C. No significant differences in HLA-DP, DQ, and DR were observed between BDA and inactive BD (BDI) patients (Supplementary Figure S2).

### 2.3. Differential Frequencies of CCR7+ Cells between Active BD and Healthy Controls

**Figure 2:**
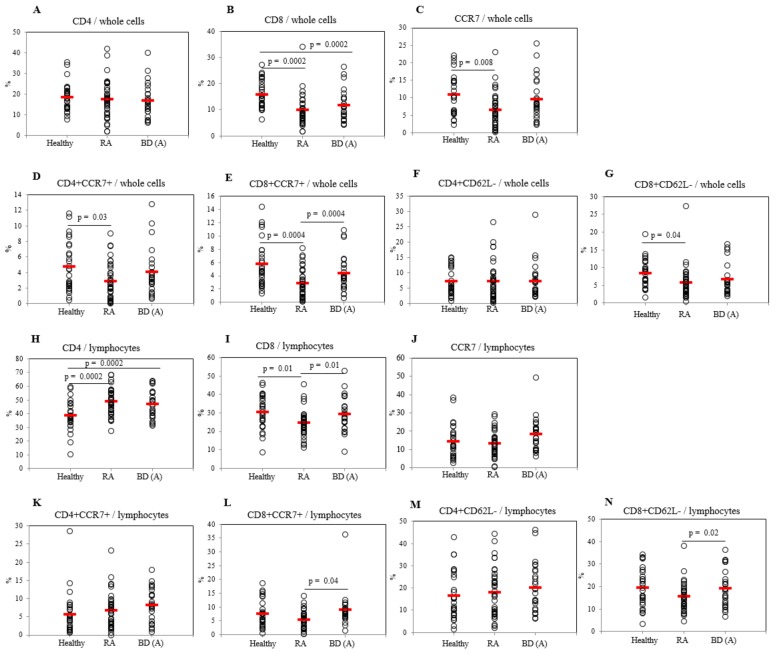
Frequencies of CD4+ (**A**) and CD8+ (**B**) cells with CCR7+ (**C–E**), CD62L- (**F,G**) cells in whole cells and in lymphocytes (**H–N**) of healthy controls (HC), rheumatoid arthritis (RA), and active Behçet’s disease (BDA) patients. Isolated peripheral blood leukocytes (PBL) were subjected to FACS analysis by surface staining. Results were obtained from 28 HC, 36 patients with RA, and 25 patients with BDA.

**Figure 3:**
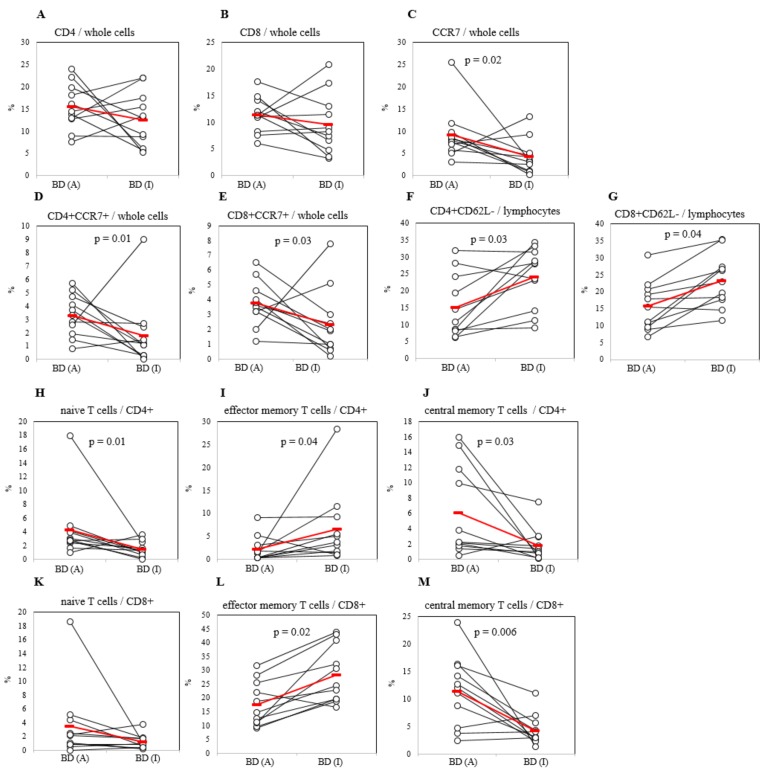
Frequencies of CD4+ and CD8+ cells with CCR7+ in whole cells (**A–E**), and with CD62L- cells in lymphocytes (**F,G**) of active Behçet’s disease (BDA) and inactive BD (BDI) patients. Frequencies of naïve T, effector memory T, central memory T cell subsets in CD4+ T cell subset (**H–J**), and in CD8+ T cell subset (**K–M**) of active Behçet’s disease (BDA) and inactive BD (BDI) patients. Isolated peripheral blood leukocyte (PBL) cells were subjected to FACS analysis by surface staining. Results were obtained from active stage and inactive stage of 11 BD patients. The *P*-value was determined by Wilcoxon rank-sum test.

Frequencies of CD4+ T cells in lymphocytes were significantly increased in BDA (46.90 ± 10.12%, *p* = 0.0002) and in RA patients (48.73 ± 9.62%, *p* = 0.0002) compared to those of HC (38.43 ± 10.83%) (Figure 2H), however, in whole cells no significant differences were found (Figure 2A). CD8+ T cells in BDA patients (11.65 ± 5.96%, *p* = 0.0002) and in RA patients (9.75 ± 5.78%, *p* = 0.0002) were significantly decreased compared to those in HC (15.65 ± 5.35%) in PBL whole cells (Figure 2B). In lymphocytes, the frequencies of CD8+ T cells of BDA patients (29.20 ± 9.50%, *p* = 0.01) were higher than those of RA patients (24.41 ± 7.20%), but not different from HC (30.20 ± 9.28%) (Figure 2I). The frequencies of CCR7+ cells in whole PBL of RA patients were significantly lower (6.38 ± 4.96%, *p* = 0.008) than those of HC (10.82 ± 5.77%), in contrast, in lymphocytes there were no significant differences among the groups (Figure 2C,J). CCR7+ cells in PBL whole cells were significantly decreased in BDI (4.31 ± 3.85%, *p* = 0.02) than those of BDA patients (9.10 ± 5.94%) (Figure 3C).

Frequencies of CD4+CCR7+ cells in PBL whole cells of RA patients (2.87 ± 2.32%, *p* = 0.03) were significantly low compared with HC (4.71 ± 3.16%), wherein lymphocytes no significant differences were observed (Figure 2D,K). In PBL whole cells, the frequencies of CD4+CCR7+ cells were significantly lower after the improvement in BDI (1.80 ± 2.54%, *p* = 0.01) compared with BDA patients (3.30 ± 1.55%) (Figure 3D). The frequencies of CD8+CCR7+ expressing cells in whole PBL of BDA patients (4.35 ± 2.75%, *p* = 0.0004) were significantly higher than those of RA patients (2.88 ± 2.20%, *p* = 0.0004), but not different to HC (5.73 ± 3.49%) (Figure 2E). CD8+CCR7+ cells were significantly decreased after improvement in BDI (2.33 ± 2.82%, *p* = 0.03) than those of BDA (3.75 ± 1.49%) patients (Figure 3E). In lymphocytes, CD8+CCR7+ expressing cells of BDA patients (8.87 ± 6.26%, *p* = 0.04) were significantly higher than those of RA patients (5.42 ± 3.21%) (Figure 2L). There were no significant differences observed in CD4+CD62L- cells frequencies among BDA, RA, and HC in PBL whole cells and in lymphocytes (Figure 2F,M). The frequencies of CD8+CD62L- cells in PBL whole cells of RA patients were lower (5.70 ± 4.60%, *p* = 0.04) than HC (8.37 ± 3.86%), in contrast, lymphocytes of BDA patients showed higher frequencies of CD8+CD62L- cells (19.28 ± 8.23%, *p* = 0.02) than those in RA patients (15.56 ± 6.32%) (Figure 2G,N). CD4+CD62L- and CD8+CD62L- cells in lymphocytes of BDI patients were increased compared with BDA patients (*p* = 0.03 and *p* = 0.03 respectively) (Supplementary Figure S3).

### 2.4. Differential Frequencies of Memory T Cells between Active and Inactive BD Patients

There were no significant differences between BDA and BDI patients in CD4+ and CD8+ T cells in whole PBL (Figure 3A,B). Whereas in lymphocytes population, the proportion of CD4+CD62L- cells in BDA patients (15.17 ± 9.26%, *p* = 0.03) were significantly lower than those in BDI (24.09 ± 8.88%) (Figure 3F); the frequencies of CD8+CD62L- cells in BDA (15.75 ± 7.24%, *p* = 0.04) were also significantly lower than those in BDI (23.25 ± 7.79%) patients (Figure 3G). In CD4+ population, frequencies of CD4+ naïve T cells of BDA patients were significantly higher than those of BDI patients (4.27 ± 4.64% *vs.* 1.48 ± 1.11%, *p* = 0.01) (Figure 3H). Frequencies of CD4+ effector memory T cells in CD4+ population were lower in BDA than those of BDI patients (2.19 ± 2.77% *vs.* 6.54 ± 8.01%, *p* = 0.04) (Figure 3I). The frequencies of CD4+ central memory T cells in CD4+ population of BDA patients were significantly higher than those of BDI patients (6.06 ± 5.87% *vs.* 1.78 ± 2.14%, *p* = 0.03) (Figure 3J). In CD8+ population, frequencies of CD8+ naïve T cells were not significant between BDA and BDI groups (Figure 3K). CD8+ effector memory T cells in BDA patients had significantly lower frequencies than those in BDI patients (17.62 ± 8.08% *vs* 28.37 ± 10.29%, *p* = 0.02) (Figure 3L). Frequencies of CD8+ central memory T cells in CD8+ population of BDA patients were significantly higher than those of BDI patients (11.41 ± 6.33% *vs* 4.26 ± 2.77%, *p* = 0.006) (Figure 3M). No significant differences were observed in naïve, effector memory, and central memory T cells among HC, RA, and BDA patients (Supplementary Figure S1).

### 2.5. Correlation between Frequencies of HLA Class II Subtypes Expressing Cells and Disease Activity Markers in BD Patients

Results of correlations between the level of disease activity markers and HLA class II subtype positive cells in BD patients are shown in Table 3. Frequencies of HLA-DQ presenting cells from whole PBL were negatively correlated with serum C-reactive protein (CRP) level (r = −0.438, *p* = 0.029). In monocytes, HLA-DR expressing cell frequencies were positively correlated with serum CRP level (r = 0.385, *p* = 0.058). HLA–DR in whole PBL was negatively correlated with erythrocyte sedimentation rate (ESR) (r = −0.330, *p* = 0.059).

### 2.6. Correlation between Frequencies of CCR7 Expressing Cells and Disease Activity Markers in BD Patients

Results of correlations between frequencies of CCR7+ T cells and disease activity markers in whole PBL and lymphocytes are shown in Table 4. The frequencies of CCR7+ cells in whole PBL were positively correlated with leukocyte number (r = 0.431, *p* = 0.031). Frequencies of CD8+CC7+ cells in whole PBL were positively correlated with leukocytes (r = 0.417, *p* = 0.038) but negatively correlated with ESR (r = -0.391, *p* = 0.053).

Frequencies of CD8+ naïve T cell in whole PBL were correlated with systemic disease score (r = 0.463, *p* = 0.02) (Table 5). In lymphocytes, frequencies of CD8 naïve T cells and systemic disease score showed significant correlation (r = 0.457, *p* = 0.022) (Table 5).

## 3. Discussion

Although the etiopathogenesis of BD is currently unknown, increased monocyte, granulocyte, and lymphocyte abnormalities have been considered to be correlated with the pathogenesis of BD [16]. Also, a strong association between HLA and autoimmune disease has long been established. This study presented significant differential frequencies of HLA class II subtypes and T cell subsets in BD compared to controls. 

HLA class II has been associated with genetic predisposition in several autoimmune diseases including RA, Grave’s disease, ankylosing spondylitis, systemic lupus erythematosus, and inflammatory bowel disease [17]. HLA-DP is a cell receptor for foreign and self-antigens. The first reported function of HLA-DP was on the presentation of herpes simplex viral antigen [18]. Viral peptide bound to DP can elicit CD4+ T cell responses [19]. HLA-DP also plays an important role in alloimmune response [20]. In juvenile rheumatoid arthritis patients, genetic polymorphism of HLA-DPw2 was significantly increased compared to that in controls [21]. In multiple sclerosis, but not in RA, the percentage of HLA-DP positive cells in monocytes was significantly lower than normal [22]. In BD patients with ocular symptoms, the association of HLA-DP antigens was not detected by polymerase chain reaction-restriction fragment length polymorphism (PCR-RFLP) methods [23]. In our present study, protein expression by flow cytometric analysis also did not show significant difference among HC, RA, and BD. There was no difference between BDA and BDI patients. The difference of HLA-DP presenting cell frequencies have specific roles in various inflammatory diseases, however, in BD, our data showed the role of HLA-DP was not significant. 

HLA-DQ can recognize and present antigens, thus stimulate T cells [24], and also have a role of a suppressor for T cell population [24,25]. It has been reported HLA-DQ molecules play a provocative role in joint destruction susceptibility of RA [26]. *HLA-DQA* and *HLA-DQB* locus are important for susceptibility of arthritis in rat models [27] and HLA-DQ8 is a highly susceptible gene in transgenic mice [28]. Whereas HLA-DR shows permissive or protective roles in mice models [29]. In BD patients with ocular symptoms, the frequencies of DQw1, DQA1*0103, DQB1*0501, and DQB1*0601 were significantly lower compared to healthy controls by PCR-RFLP [30]. On the other hand, in BD patients with cutaneous symptoms, the frequencies of DQw6 were significantly higher than healthy controls [31]. Our data, in BD patients with arthritis, according to flow cytometric analysis by staining for cell surface protein expression showed frequencies of HLA-DQ positive cells were significantly decreased compared to healthy controls in whole cells and in granulocytes. In RA patients, HLA-DQ positive cells also showed significant lower frequencies than healthy controls. There were no significant differences between RA and BD with arthritis in HLA-DQ positive cell frequencies. We can speculate HLA-DQ surface expressing cell portions play an important role for pathogenesis in arthritis including BD arthritis. HLA-DQ positive cell frequencies were not changed after arthritis improvement in BD. This means HLA-DQ expressing cell population is correlated to the induction of BD, however, HLA-DQ expressing cell population does not influence the improvement of arthritis symptoms.

Low expression of HLA-DR molecules in monocytes is a risk factor of infection [32]. HLA-DR molecules also present self-antigens to T cells. They can induce an inflammatory response which subsequently induces arthritis in patients [29]. Uveitis patients, including BD uveitis, show higher frequencies of HLA-DR than healthy controls by flow cytometric analysis [33]. Lehner et al. reported HLA-DR2 and DR7 were correlated to BD by the lymphocytotoxicity test [34]. According to PCR-RFLP analysis, the frequencies of DRB1*0802 were significantly higher in BD patients with ocular symptoms than healthy controls [30]. In BD patients with cutaneous symptoms, HLA-DRB1*14 was significantly increased and HLA-DRB1*15 was significantly decreased [35]. In our study, by flow cytometric analysis, the frequencies of HLA-DR expressing cells were significantly higher in BD than healthy controls in monocytes population. After improvement of arthritis in BD, the frequencies of HLA-DR expressing cells in monocytes population were not significantly changed. This means HLA-DR positive monocytes were not directly correlated to the improvement of arthritis symptom in BD.

CC-chemokine receptor 7 (CCR7) has a pathogenic role in Crohn’s disease [36]. In inflammatory bowel disease, blockage of CCR7 has been suggested as a target for treatment [37]. In multiple sclerosis, deficiency of effector memory CD8+ T cells has been observed from an early stage [38]. CCR7 and CD62L have been applied to categorize human memory T cells into functionally distinct subsets [39], and are key players for homing of naïve T cells to lymph nodes and Peyer’s patches [40,41]. Based on the expression of CCR7 and CD62L, memory T cells can be subdivided into central memory and effector memory cells [42]. In RA patients, the population of CD45RA-CD62L+CD8+ central memory T cells was increased in peripheral blood whereas the population of CD45RA+CD62L-CD8+ effector memory T cells was decreased [43]. In BD patients with arthritis in our data, the frequencies of CCR7-CD62L-CD4+, CCR7-CD62L-CD8+ effector memory T cells, and CCR7+CD62L+CD4+, CCR7+CD62L+CD8+ central memory T cells were not different compared to HC or RA in PBL whole cells and in lymphocytes populations. RA patients showed similar frequencies of CCR7-CD62L-CD4+, CCR7-CD62L-CD8+ effector memory T cells, and CCR7+CD62L+CD4+, CCR7+CD62L+CD8+ central memory T cells compared to HC. On the other hand, in the CD4+ population, the proportion of CCR7-CD62L- effector memory T cells were increased, but CCR7+CD62L+ central memory T cells were decreased after arthritis improvement in BD patients. In the CD8+ population, the changes of frequencies showed similar tendencies with CD4+ population. 

Ellingsen et al. have reported that increased CD4+CCR7+ expression on surface of peripheral monocytes in RA patients was normalized after treatment [44]. With the same tendency, in BD patients with arthritis, CCR7+, CD4+CCR7+, and CD8+CCR7+ cell frequencies in whole cells were decreased after improvement. CD62L controls the migration of T cells and plays an important role in shedding at the cell membrane after T cell activation [45]. CD4+CD62L- and CD8+CD62L- cell proportions in whole PBL population were decreased in adult-onset Still’s disease (AOSD) compared to RA or healthy controls [46]. CD4+CD62L- and CD8+CD62L- cell proportions of AOSD were not changed after improvement, even though AOSD also showed arthritis. In our study of BD patients with arthritis, CD4+CD62L- and CD8+CD62L- cells in lymphocyte population of active BD patients were significantly increased after improvement compared to the same group of inactive BD patients. According to Maldonado et al., frequencies of CD45RA-CD62L+CD4+ and CD45RA-CD62L+CD8+ central memory T cells were increased in RA patients and this upregulation may have accelerated the maturation of naïve T cells [43]. In our study, we also found CD4 + and CD8 + central memory T cells were increased in active BD patients, and CD4 + naïve T cells were also increased, which were decreased in inactive BD patients, suggesting an increased number of central memory T cells may have a role in the pathogenesis of BD. In RA patients, the numbers of CD45RA+ CD62L- effector memory CD8 + T cells were reduced in the peripheral blood [43]. Similarly, in our results, CD4+ and CD8+ effector memory T cells in active BD patients were decreased and increased in inactive BD patients through improvement.

Our study revealed HLA class II subtypes DP, DQ, and DR molecule presenting cells on PBL and proportions of circulating T cell phenotypes in patients with BD arthritis according to presence of symptoms. Active, untreated BD patients have decreased HLA-DQ levels in whole cells and in granulocytes. HLA-DR levels were increased in monocytes of untreated active BD patients. HLA-DR in monocytes was positively correlated with serum CRP levels, whereas HLA-DQ frequencies showed negative correlation with CRP levels. However, the proportions of HLA subtypes were not significantly changed after improvement of BD arthritis. CCR7 positive cells were decreased after improvement and positively associated with blood leukocyte levels, and CD4+CCR7+ and CD8+CCR7+ in whole PBL, CD4+CD62L- and CD8+CD62L- in lymphocytes population were correlated to the presence of arthritis symptoms of BD. Naïve T cells and central memory T cells were decreased after improvement, however, effector memory T cell frequencies were increased after improvement suggesting upregulation of central memory T cells and decreased of effector memory T cells were related to disease progression. Results of this study specifically indicate HLA-DP, DQ, and DR expressing cell frequencies and several T cell subsets are significantly correlated with BD arthritis symptoms.

## 4. Materials and Methods

### 4.1. Patients

Patients with BD were diagnosed according to the International Study Group of Behçet’s Disease [47]. BD activity index was calculated as outlined in the BD Current Activity Form 2006 (http://medhealth.leeds.ac.uk/download/910/behcetsdiseaseactivityform). Disease was evaluated following a consultant-led assessment at the time of collecting sampling. Patients were defined as active BD (BDA) if they presented with two or more of the following disease manifestations: oral aphthous ulcer, genital ulcers, positive pathergy test, skin lesions, ocular, vascular and neurological involvement, and arthritis. Blood sampling was done at active stage and inactive stage (BDI) during follow-up after improvement in joint symptoms. There were 25 patients (5 males, 20 females, mean age of 48.8 ± 7.6 years) in the group of BDA with arthritis and 11 patients in the group of BDI. As disease control, RA patients (*n* = 36, 11 males, 25 females, mean age of 30.4 ± 10.1 years) were enrolled. Rheumatoid arthritis (RA) patients satisfied the American College of Rheumatology 1987 revised criteria for the classification of RA [48]. Healthy individuals without history of autoimmune, rheumatic, or any other disease (*n* = 28, 10 males, 18 females, mean age of 38.4 ± 15.8 years) were included in the healthy control (HC) group. Patients were recruited from the Department of Rheumatology at Ajou University Hospital, Republic of Korea. Medical histories and clinical characteristics of all subjects were collected via a review of subjects’ medical records and an interview with the subject when samples were collected. Clinical characteristics of BD patients and medication to patients are presented in Table 1 and Table 2. In RA patients, blood sampling was done during medication. Medication for RA patients is shown in Supplementary Table S2. Enrolled HC, RA, and BD in this study were also partly used in our previous study and analyzed with different markers [49]. This study was approved by the Institutional Review Board of Ajou University Hospital [approval number: AJIRB-BMR-SMP-13-398, Project code 2013R1A1A3008248 (01 June 2013)]. All subjects were recruited with signed informed consent. 

### 4.2. Flow Cytometry Analysis of Patients’ Blood Samples

Peripheral blood cells were treated with Ammonium-Chloride-Potassium solution to lyse red blood cells and washed with phosphate buffered saline (PBS). Washed cells (1 × 10^6^) in each tube were incubated with, PE-labeled anti-human HLA-DR, FITC-labeled anti-human HLA-DQ, APC-Cy7-labeled anti-human CD4, PE-labeled anti-human CCR7, PerCP-eFluro 710-labeled anti-human CD62L, PE-Cy7-labeled anti-human CD45RO, APC-labeled anti-human CD45RA (eBioscience, San Diego, CA, USA), FITC-labeled anti-human HLA-DP (Abbiotec. LLC, San Diego, CA 92126, USA), FITC-labeled anti-Human CD8 (BD PharMingen, San Jose, CA 95131, USA), for 30 min at 4 °C. The same color tagged antibodies were applied to different tubes. Stained cells were washed with PBS and analyzed with a flow cytometer (FACS Aria III; Becton Dickinson, San Jose, CA, USA) using at least 10,000 cells. Naïve T cell marker was CD45RO-CD45RA+CCR7+CD62L+ in combination with CD4+ or CD8+. Effector memory T cell marker was CD45RO+CD45RA-CCR7-CD62L- in combination with CD4+ or CD8+. Central memory T cell marker was CD45RO+CD45RA-CCR7+CD62L+ in combination with CD4+ or CD8+. FACS data were collected based on gating of whole cells, granulocytes, monocytes, and lymphocytes. Specific markers were used to analyze gated population. 

### 4.3. Statistical Analysis

The frequencies of cellular markers among healthy control, RA, and BD were compared using analysis of variance (ANOVA) test. The difference between BDA and BDI was examined with Wilcoxon rank-sum test. All data were described as mean ± standard deviation for parametric test (i.e., ANOVA) and as median ± interquartile range (IQR) for nonparametric test (i.e., Wilcoxon test). Statistical analyses were performed using IBM Statistical Package for the Social Sciences (IBM SPSS, version 23.0, Armonk, NY, USA) software. A threshold value of *p* < 0.05 was considered as statistical significance.

## 5. Conclusions

Our study revealed HLA-DQ presenting cells were decreased in BD patients with or without arthritic symptoms when compared to healthy controls. The proportions of HLA-DP, DQ, and DR presenting cells were not significantly changed after improvement of BD arthritis. Therefore, the lower proportion of HLA-DQ presenting cells can be a triggering factor of BD arthritis. The frequencies of effector memory T cells and central memory T cells were significantly changed after improvement of BD arthritis and were controlled in reverse between effector memory T and central memory T cells. We can conclude HLA-DQ presenting cell frequencies and memory T subsets are significantly correlated with BD arthritis symptoms.

## Figures and Tables

**Table 1:** Clinical characteristics of patients with Behçet’s disease at blood sampling.

Patients	Age	OU	GU	Arth	EN	Disease Severity Score		Leukocyte		ESR		CRP	
						1st	2nd	1st	2nd	1st	2nd	1st	2nd
N = 25 (M = 5, F = 20)	48.8 ± 7.6	13 (52.0%)	6 (24.0%)	25 (100%)	7 (28.0%)	3.04 ± 1.1	1.27 ± 1.0	8008.0 ± 3480.1	6836.4 ± 1937.1	29.8 ± 25.8	18.3 ±13.6	1.01 ± 2.02	0.26 ± 0.37
*P* value						*P* = 0.007		*P* = 0.168		*P* = 0.083		*P* = 0.314	

**Note**—M: male, F: female, OU: oral ulcers, GU: genital ulcers, Arth: arthritis, EN: erythema nodosum, Score: Disease severity score, ESR: erythrocyte sedimentation rate, CRP: C-reactive protein, 1st: first blood sampling (*n* = 25), 2nd: second blood sampling (*n* = 11).

**Table 2:** Medication for patients with Behçet’s disease at blood sampling.

Ordrer	Colchicine		Steroid		AZP		Bucillamine		HCQ		SZP		NSAIDs	
	1st	2nd	1st	2nd	1st	2nd	1st	2nd	1st	2nd	1st	2nd	1st	2nd
Number (%)	19 (76.2%)	9 (81.8%)	21 (84.0%)	7 (63.6%)	7 (28.0%)	3 (27.3%)	1 (8.0%)	2 (18.2%)	8 (32.0%)	4 (36.4%)	10 (40.0%)	5 (45.5%)	16 (64.0%)	7 (63.6%)

**Note**—AZP: Azathioprine, HCQ: Hydroxychloroquine, SZP: Sulphasalazine, NSAIDs: Nonsteroidal Anti-Inflammatory Drugs, 1st: first blood sampling (*n* = 25), 2nd: second blood sampling (*n* = 11).

**Table 3:** Correlations between the frequencies of HLA-DP, DQ, and DR positive cells and disease activity markers.

Disease Activity Markers	Correlation Coefficient, r (*p*-value)												
	HLA-DP				HLA-DQ				HLA-DR			
	PBL	Monocyte	Lymphocyte	Granulocyte	PBL	Monocyte	Lymphocyte	Granulocyte	PBL	Monocyte	Lymphocyte	Granulocyte
Disease severity score	−0.015 (0.945)	0.202 (0.334)	0.138 (0.510)	0.193 (0.355)	−0.033 (0.876)	0.365 (0.073)	0.091 (0.665)	−0.088 (0.677)	−0.004 (0.984)	0.379 (0.062)	0.374 (0.066)	−0.041 (0.847)
Leukocyte number	0.025 (0.907)	−0.226 (0.278)	−0.169 (0.419)	0.123 (0.557)	−0.021 (0.919)	−0.323 (0.115)	−0.394 (0.051)	0.364 (0.073)	0.218 (0.294)	−0.104 (0.622)	−0.117 (0.579)	0.289 (0.161)
ESR	−0.142 (0.498)	0.044 (0.834)	−0.289 (0.161)	0.180 (0.389)	−0.225 (0.279)	0.055 (0.792)	0.010 (0.964)	−0.230 (0.269)	−0.33 (0.059)	0.107 (0.610)	0.060 (0.777)	−0.034 (0.872)
CRP	−0.179 (0.393)	0.213 (0.306)	−0.289 (0.161)	0.001 (0.996)	−0.438 (0.029)	−0.213 (0.307)	−0.290 (0.160)	−0.078 (0.709)	−0.234 (0.260)	0.385 (0.058)	−0.005 (0.982)	−0.062 (0.769)

**Note**—PBL, peripheral blood leukocytes; ESR, erythrocyte sedimentation rate; CRP, C-reactive protein.

**Table 4:** Correlations between the frequencies of T cells and disease activity markers.

Disease Activity Markers	Correlation Coefficient, r (*p*-value)						
	CD4+	CD8+	CCR7+	CD4+CCR7+	CD8+CCR7+	CD4+CD62L-	CD8+CD62L-
Leukocyte number	0.316 (0.124)	0.378 (0.063)	0.431 (0.031)	0.351 (0.086)	0.417 (0.038)	0.325 (0.113)	0.266 (0.198)
ESR	−0.382 (0.060)	−0.376 (0.064)	−0.258 (0.213)	−0.385 (0.057)	−0.391 (0.053)	−0.267 (0.197)	−0.339 (0.097)
CRP	−0.161 (0.442)	−0.198 (0.342)	−0.119 (0.572)	−0.187 (0.370)	−0.184 (0.380)	−0.063 (0.764)	−0.109 (0.603)

**Note**—ESR, erythrocyte sedimentation rate; CRP, C-reactive protein.

**Table 5:** Correlations between the frequencies of memory T cells and disease severity score.

	Correlation Coefficient, r (*p*-value)					
	CD4+ Naïve T	CD4+ Effector Memory T	CD4+ Central Memory T	CD8+ Naïve T	CD8+ Effector Memory T	CD8+ Central Memory T
Disease Severity Score	0.259 (0.212)	−0.358 (0.079)	0.075 (0.723)	0.463 (0.020)	−0.214 (0.305)	0.163 (0.435)

## Supplementary Materials

Supplementary materials can be found at https://www.mdpi.com/1422-0067/20/20/5044/s1.
