# Supplementary material for: Differences in Expression of Human Leukocyte Antigen Class II Subtypes and T Cell Subsets in Behçet’s Disease with Arthritis
Source: Int J Mol Sci. 2019 Oct 11;20(20):5044. doi: 10.3390/ijms20205044 (PMC6829274; doi:10.3390/ijms20205044)
Supplement: Supplementary file 1 [file ijms-20-05044-s001.zip › supplementary/Supplementary Figures.pptx]

## Slide 1
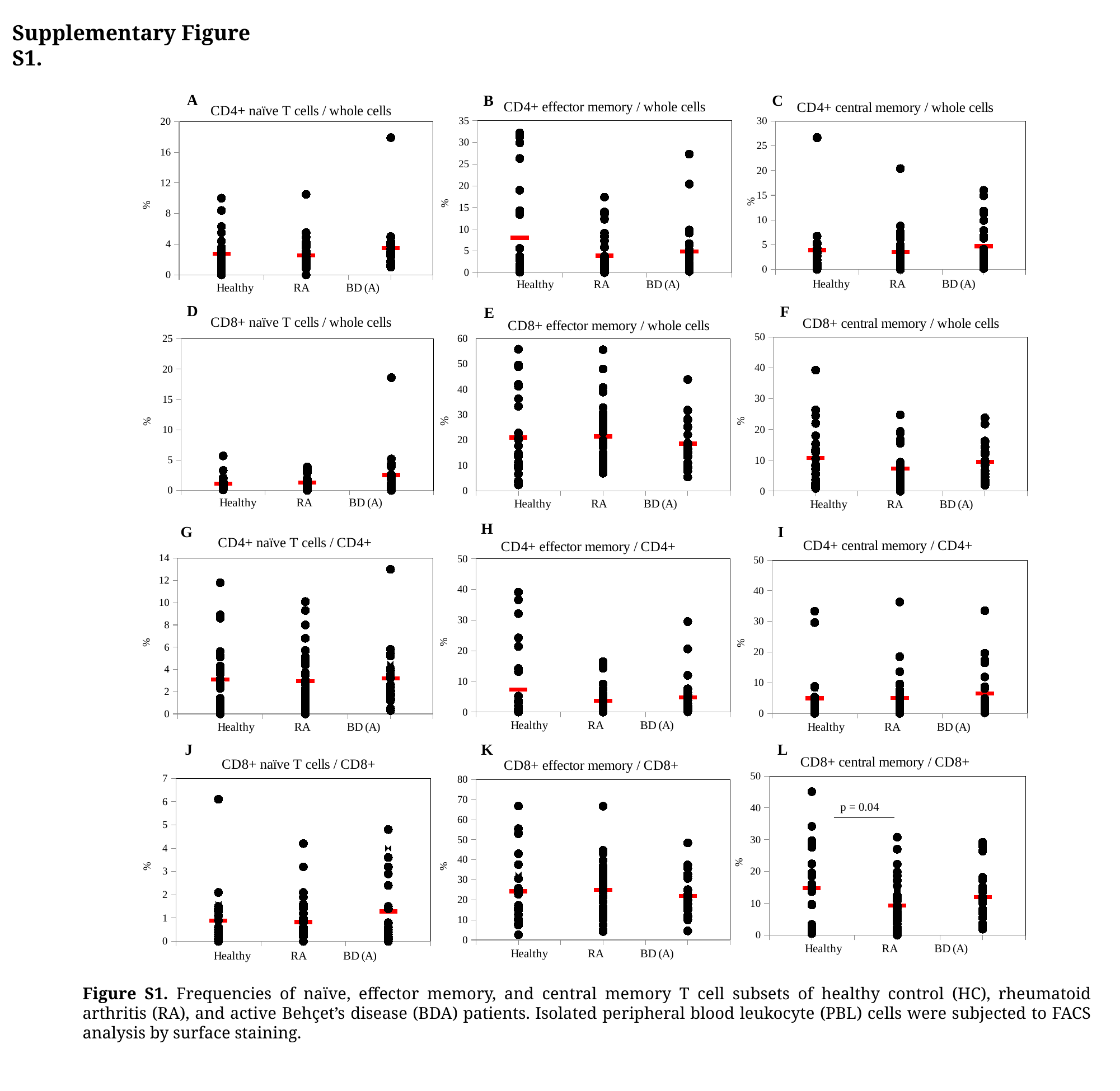

Supplementary Figure S1.
### Chart: CD4+ central memory / whole cells
| Category | | | | | | | | | | | | | | | | | | | | | | | | | | | | | | | | | | | | | | | | | | | |
|---|---|---|---|---|---|---|---|---|---|---|---|---|---|---|---|---|---|---|---|---|---|---|---|---|---|---|---|---|---|---|---|---|---|---|---|---|---|---|---|---|---|---|---|
### Chart: CD4+ effector memory / whole cells
| Category | | | | | | | | | | | | | | | | | | | | | | | | | | | | | | | | | | | | | | | | | | | |
|---|---|---|---|---|---|---|---|---|---|---|---|---|---|---|---|---|---|---|---|---|---|---|---|---|---|---|---|---|---|---|---|---|---|---|---|---|---|---|---|---|---|---|---|
### Chart: CD4+ naïve T cells / whole cells
| Category | | | | | | | | | | | | | | | | | | | | | | | | | | | | | | | | | | | | | | | | | | | |
|---|---|---|---|---|---|---|---|---|---|---|---|---|---|---|---|---|---|---|---|---|---|---|---|---|---|---|---|---|---|---|---|---|---|---|---|---|---|---|---|---|---|---|---|A
B
C
### Chart: CD8+ effector memory / whole cells
| Category | | | | | | | | | | | | | | | | | | | | | | | | | | | | | | | | | | | | | | | | | | | |
|---|---|---|---|---|---|---|---|---|---|---|---|---|---|---|---|---|---|---|---|---|---|---|---|---|---|---|---|---|---|---|---|---|---|---|---|---|---|---|---|---|---|---|---|D
F
E
### Chart: CD8+ central memory / whole cells
| Category | | | | | | | | | | | | | | | | | | | | | | | | | | | | | | | | | | | | | | | | | | | |
|---|---|---|---|---|---|---|---|---|---|---|---|---|---|---|---|---|---|---|---|---|---|---|---|---|---|---|---|---|---|---|---|---|---|---|---|---|---|---|---|---|---|---|---|
### Chart: CD8+ naïve T cells / whole cells
| Category | | | | | | | | | | | | | | | | | | | | | | | | | | | | | | | | | | | | | | | | | | | |
|---|---|---|---|---|---|---|---|---|---|---|---|---|---|---|---|---|---|---|---|---|---|---|---|---|---|---|---|---|---|---|---|---|---|---|---|---|---|---|---|---|---|---|---|H
### Chart: CD4+ effector memory / CD4+
| Category | | | | | | | | | | | | | | | | | | | | | | | | | | | | | | | | | | | | | | | | | | | | | | |
|---|---|---|---|---|---|---|---|---|---|---|---|---|---|---|---|---|---|---|---|---|---|---|---|---|---|---|---|---|---|---|---|---|---|---|---|---|---|---|---|---|---|---|---|---|---|---|G
I
### Chart: CD4+ central memory / CD4+
| Category | | | | | | | | | | | | | | | | | | | | | | | | | | | | | | | | | | | | | | | | | | | | | | |
|---|---|---|---|---|---|---|---|---|---|---|---|---|---|---|---|---|---|---|---|---|---|---|---|---|---|---|---|---|---|---|---|---|---|---|---|---|---|---|---|---|---|---|---|---|---|---|
### Chart: CD4+ naïve T cells / CD4+
| Category | | | | | | | | | | | | | | | | | | | | | | | | | | | | | | | | | | | | | | | | | | | | | | |
|---|---|---|---|---|---|---|---|---|---|---|---|---|---|---|---|---|---|---|---|---|---|---|---|---|---|---|---|---|---|---|---|---|---|---|---|---|---|---|---|---|---|---|---|---|---|---|L
K
J
### Chart: CD8+ naïve T cells / CD8+
| Category | | | | | | | | | | | | | | | | | | | | | | | | | | | | | | | | | | | | | | | | | | | | | | |
|---|---|---|---|---|---|---|---|---|---|---|---|---|---|---|---|---|---|---|---|---|---|---|---|---|---|---|---|---|---|---|---|---|---|---|---|---|---|---|---|---|---|---|---|---|---|---|
### Chart: CD8+ central memory / CD8+
| Category | | | | | | | | | | | | | | | | | | | | | | | | | | | | | | | | | | | | | | | | | | | | | | |
|---|---|---|---|---|---|---|---|---|---|---|---|---|---|---|---|---|---|---|---|---|---|---|---|---|---|---|---|---|---|---|---|---|---|---|---|---|---|---|---|---|---|---|---|---|---|---|
### Chart: CD8+ effector memory / CD8+
| Category | | | | | | | | | | | | | | | | | | | | | | | | | | | | | | | | | | | | | | | | | | | | | | |
|---|---|---|---|---|---|---|---|---|---|---|---|---|---|---|---|---|---|---|---|---|---|---|---|---|---|---|---|---|---|---|---|---|---|---|---|---|---|---|---|---|---|---|---|---|---|---|p = 0.04
Figure S1. Frequencies of naïve, effector memory, and central memory T cell subsets of healthy control (HC), rheumatoid arthritis (RA), and active Behçet’s disease (BDA) patients. Isolated peripheral blood leukocyte (PBL) cells were subjected to FACS analysis by surface staining.

## Slide 2
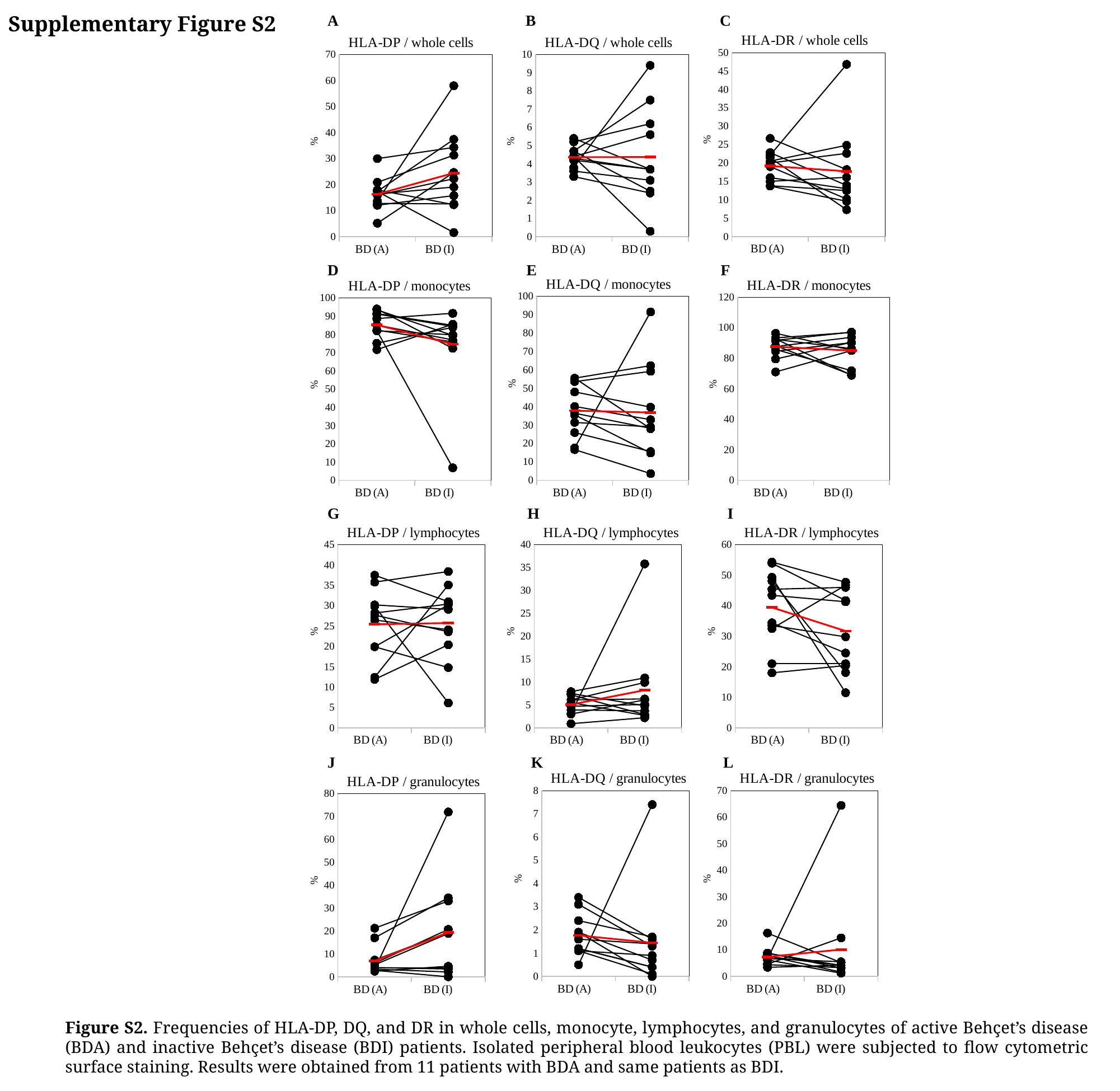

Supplementary Figure S2
A B C
### Chart: HLA-DR / whole cells
| Category | | | | | | | | | | | | |
|---|---|---|---|---|---|---|---|---|---|---|---|---|
| BD (A) | 20.5 | 13.8 | 26.7 | 16.0 | 22.8 | 15.0 | 21.4 | 22.1 | 19.0 | 13.7 | 20.0 | 19.18181818181818 |
| BD (I) | 24.8 | 12.5 | 18.2 | 13.0 | 13.9 | 16.1 | 7.3 | 46.8 | 10.2 | 9.6 | 22.6 | 17.727272727272723 |
### Chart: HLA-DP / whole cells
| Category | | | | | | | | | | | | |
|---|---|---|---|---|---|---|---|---|---|---|---|---|
| BD (A) | 16.4 | 5.2 | 18.0 | 15.9 | 17.4 | 12.1 | 30.0 | 17.4 | 12.8 | 20.9 | 13.7 | 16.345454545454544 |
| BD (I) | 19.1 | 24.7 | 12.3 | 22.3 | 1.6 | 15.8 | 34.3 | 37.4 | 12.6 | 31.3 | 58.0 | 24.49090909090909 |
### Chart: HLA-DQ / whole cells
| Category | | | | | | | | | | | | |
|---|---|---|---|---|---|---|---|---|---|---|---|---|
| BD (A) | 4.4 | 3.3 | 4.2 | 4.3 | 3.8 | 3.6 | 5.4 | 5.2 | 4.4 | 4.7 | 4.7 | 4.363636363636364 |
| BD (I) | 5.6 | 2.4 | 3.7 | 3.7 | 9.4 | 3.1 | 3.7 | 6.2 | 0.3 | 2.5 | 7.5 | 4.372727272727272 |D E F
### Chart: HLA-DQ / monocytes
| Category | | | | | | | | | | | | |
|---|---|---|---|---|---|---|---|---|---|---|---|---|
| BD (A) | 31.4 | 25.9 | 48.0 | 53.6 | 17.6 | 35.5 | 40.1 | 55.5 | 16.6 | 55.5 | 36.4 | 37.82727272727273 |
| BD (I) | 29.0 | 15.6 | 39.7 | 59.2 | 91.5 | 14.8 | 32.9 | 62.3 | 3.6 | 28.0 | 28.2 | 36.800000000000004 |
### Chart: HLA-DR / monocytes
| Category | | | | | | | | | | | | |
|---|---|---|---|---|---|---|---|---|---|---|---|---|
| BD (A) | 84.6 | 85.7 | 92.3 | 96.3 | 87.7 | 93.3 | 88.2 | 92.7 | 91.7 | 71.0 | 79.5 | 87.54545454545456 |
| BD (I) | 90.2 | 71.8 | 86.3 | 85.3 | 93.7 | 96.9 | 69.1 | 68.8 | 97.1 | 85.0 | 90.4 | 84.96363636363637 |
### Chart: HLA-DP / monocytes
| Category | | | | | | | | | | | | |
|---|---|---|---|---|---|---|---|---|---|---|---|---|
| BD (A) | 82.4 | 82.0 | 91.2 | 94.0 | 82.1 | 93.5 | 85.2 | 91.5 | 88.7 | 71.7 | 75.2 | 85.22727272727275 |
| BD (I) | 75.9 | 79.8 | 84.4 | 72.4 | 6.9 | 79.5 | 77.0 | 85.0 | 91.6 | 85.6 | 83.9 | 74.72727272727273 |G H I
### Chart: HLA-DP / lymphocytes
| Category | | | | | | | | | | | | |
|---|---|---|---|---|---|---|---|---|---|---|---|---|
| BD (A) | 37.5 | 12.4 | 28.2 | 27.7 | 29.9 | 26.5 | 30.2 | 35.8 | 19.9 | 19.9 | 11.9 | 25.44545454545454 |
| BD (I) | 31.0 | 35.1 | 30.4 | 23.6 | 6.1 | 24.1 | 29.1 | 38.4 | 30.2 | 14.8 | 20.4 | 25.74545454545454 |
### Chart: HLA-DQ / lymphocytes
| Category | | | | | | | | | | | | |
|---|---|---|---|---|---|---|---|---|---|---|---|---|
| BD (A) | 7.9 | 0.9 | 3.0 | 6.1 | 3.1 | 6.1 | 3.9 | 4.7 | 7.2 | 7.5 | 5.5 | 5.081818181818182 |
| BD (I) | 10.9 | 2.2 | 6.1 | 9.9 | 35.8 | 6.3 | 3.7 | 5.1 | 2.9 | 4.8 | 2.7 | 8.218181818181819 |
### Chart: HLA-DR / lymphocytes
| Category | | | | | | | | | | | | |
|---|---|---|---|---|---|---|---|---|---|---|---|---|
| BD (A) | 43.4 | 54.3 | 45.4 | 53.9 | 32.5 | 33.5 | 49.3 | 48.1 | 21.0 | 34.4 | 18.0 | 39.43636363636364 |
| BD (I) | 41.3 | 47.7 | 46.0 | 41.7 | 46.7 | 29.8 | 11.5 | 18.1 | 21.0 | 24.5 | 20.4 | 31.7 |J K L
### Chart: HLA-DQ / granulocytes
| Category | | | | | | | | | | | | |
|---|---|---|---|---|---|---|---|---|---|---|---|---|
| BD (A) | 1.9 | 3.4 | 1.2 | 1.1 | 3.1 | 1.1 | 2.4 | 1.6 | 1.9 | 1.2 | 0.5 | 1.7636363636363634 |
| BD (I) | 0.7 | 1.6 | 0.4 | 0.9 | 1.3 | 0.1 | 1.7 | 1.4 | 0.0 | 0.4 | 7.4 | 1.4454545454545455 |
### Chart: HLA-DR / granulocytes
| Category | | | | | | | | | | | | |
|---|---|---|---|---|---|---|---|---|---|---|---|---|
| BD (A) | 7.5 | 16.3 | 6.7 | 3.4 | 8.7 | 4.4 | 6.2 | 6.5 | 7.7 | 8.8 | 4.6 | 7.345454545454544 |
| BD (I) | 4.1 | 5.2 | 5.5 | 4.2 | 4.1 | 3.3 | 1.2 | 64.4 | 1.6 | 3.1 | 14.5 | 10.109090909090908 |
### Chart: HLA-DP / granulocytes
| Category | | | | | | | | | | | | |
|---|---|---|---|---|---|---|---|---|---|---|---|---|
| BD (A) | 3.9 | 5.2 | 3.3 | 7.3 | 2.8 | 2.4 | 17.0 | 5.9 | 4.0 | 21.2 | 3.2 | 6.927272727272728 |
| BD (I) | 3.8 | 18.9 | 2.1 | 19.4 | 0.0 | 4.6 | 34.5 | 20.7 | 3.4 | 33.1 | 72.0 | 19.31818181818182 |Figure S2. Frequencies of HLA-DP, DQ, and DR in whole cells, monocyte, lymphocytes, and granulocytes of active Behçet’s disease (BDA) and inactive Behçet’s disease (BDI) patients. Isolated peripheral blood leukocytes (PBL) were subjected to flow cytometric surface staining. Results were obtained from 11 patients with BDA and same patients as BDI.

## Slide 3
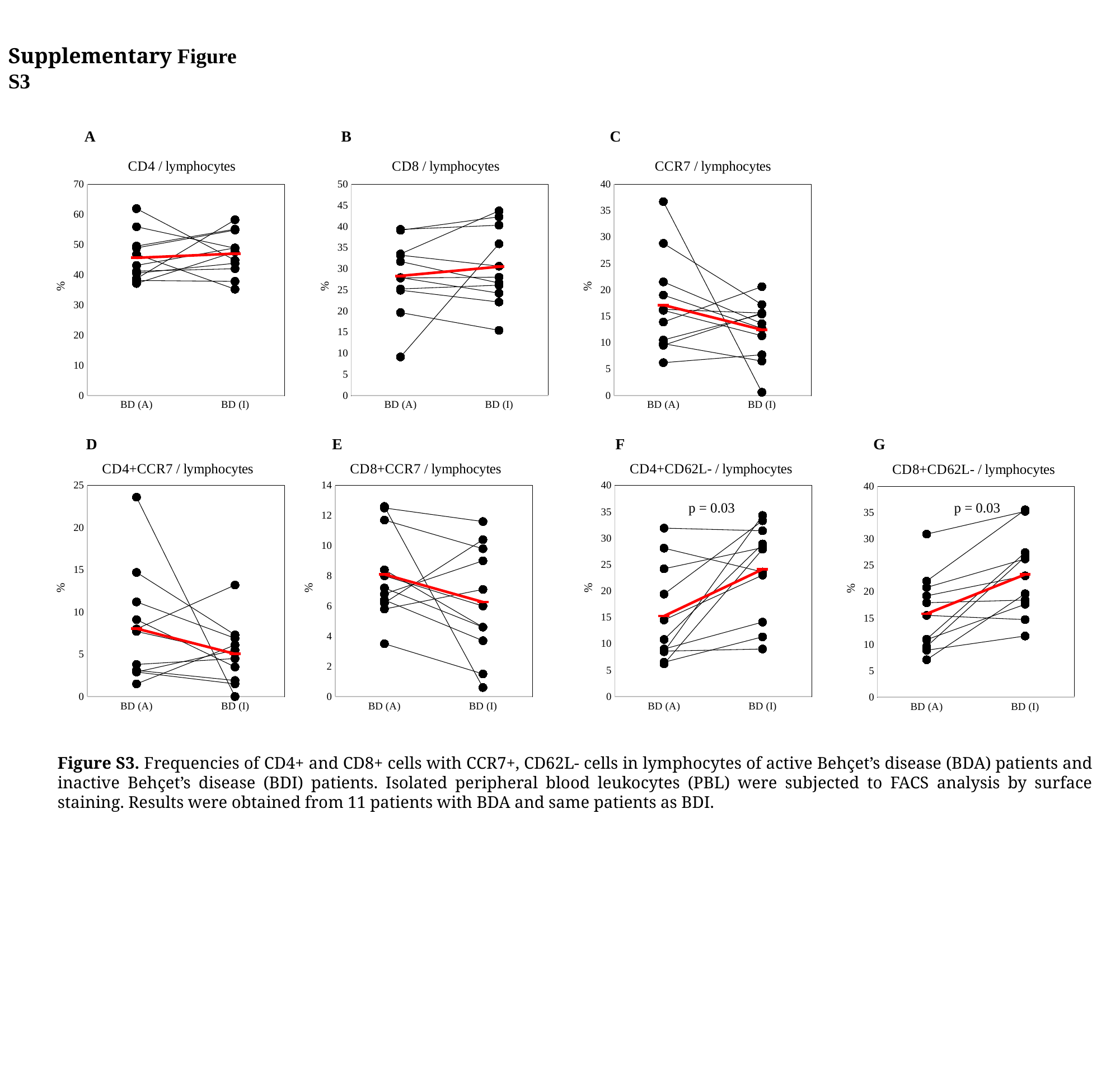

Supplementary Figure S3
A B C
### Chart: CD4 / lymphocytes
| Category | | | | | | | | | | | | |
|---|---|---|---|---|---|---|---|---|---|---|---|---|
| BD (A) | 38.1 | 38.7 | 43.1 | 46.8 | 40.5 | 48.9 | 41.2 | 37.1 | 61.9 | 49.5 | 55.9 | 45.6090909090909 |
| BD (I) | 37.8 | 58.2 | 48.9 | 35.2 | 43.8 | 54.8 | 42.0 | 47.6 | 44.8 | 55.1 | 48.8 | 47.00000000000001 |
### Chart: CD8 / lymphocytes
| Category | | | | | | | | | | | | |
|---|---|---|---|---|---|---|---|---|---|---|---|---|
| BD (A) | 31.7 | 19.6 | 33.2 | 39.1 | 24.9 | 27.9 | 39.3 | 9.1 | 27.8 | 25.2 | 33.5 | 28.3 |
| BD (I) | 26.6 | 15.4 | 30.6 | 42.3 | 22.1 | 24.2 | 40.3 | 35.9 | 28.0 | 26.1 | 43.7 | 30.472727272727273 |
### Chart: CCR7 / lymphocytes
| Category | | | | | | | | | | | | |
|---|---|---|---|---|---|---|---|---|---|---|---|---|
| BD (A) | 19.0 | 28.8 | 9.5 | 16.3 | 9.8 | 16.1 | 21.5 | 10.5 | 36.7 | 6.2 | 13.9 | 17.118181818181817 |
| BD (I) | 12.7 | 17.2 | 15.6 | 15.6 | 6.5 | 11.3 | 13.6 | 15.4 | 0.6 | 7.7 | 20.6 | 12.436363636363636 |D E 	 F	 G
### Chart: CD4+CCR7 / lymphocytes
| Category | | | | | | | | | | | | |
|---|---|---|---|---|---|---|---|---|---|---|---|---|
| BD (A) | 11.2 | 14.7 | 2.9 | 3.1 | 2.9 | 7.7 | 9.1 | 3.8 | 23.6 | 1.5 | 8.0 | 8.045454545454545 |
| BD (I) | 6.9 | 7.3 | 5.5 | 1.9 | 1.5 | 5.1 | 3.5 | 4.5 | 0.0 | 6.1 | 13.2 | 5.045454545454546 |
### Chart: CD8+CCR7 / lymphocytes
| Category | | | | | | | | | | | | |
|---|---|---|---|---|---|---|---|---|---|---|---|---|
| BD (A) | 7.2 | 8.4 | 6.8 | 12.5 | 6.4 | 8.0 | 11.7 | 6.2 | 12.6 | 3.5 | 5.8 | 8.1 |
| BD (I) | 4.6 | 4.6 | 9.0 | 11.6 | 3.7 | 6.0 | 9.8 | 10.4 | 0.6 | 1.5 | 7.1 | 6.263636363636363 |
### Chart: CD4+CD62L- / lymphocytes
| Category | | | | | | | | | | | | |
|---|---|---|---|---|---|---|---|---|---|---|---|---|
| BD (A) | 6.2 | 8.5 | 19.4 | 24.2 | 14.5 | 8.6 | 31.9 | 10.8 | 9.0 | 28.1 | 6.5 | 15.245454545454542 |
| BD (I) | 27.9 | 34.3 | 33.3 | 28.2 | 23.0 | 9.0 | 31.4 | 28.9 | 14.1 | 23.6 | 11.3 | 24.09090909090909 |
### Chart: CD8+CD62L- / lymphocytes
| Category | | | | | | | | | | | | |
|---|---|---|---|---|---|---|---|---|---|---|---|---|
| BD (A) | 19.2 | 8.9 | 20.8 | 22.0 | 10.9 | 15.5 | 30.9 | 11.0 | 7.1 | 17.9 | 9.6 | 15.8 |
| BD (I) | 23.0 | 11.6 | 26.2 | 35.5 | 17.6 | 14.7 | 35.2 | 27.4 | 19.6 | 18.4 | 26.6 | 23.254545454545454 |p = 0.03
p = 0.03
Figure S3. Frequencies of CD4+ and CD8+ cells with CCR7+, CD62L- cells in lymphocytes of active Behçet’s disease (BDA) patients and inactive Behçet’s disease (BDI) patients. Isolated peripheral blood leukocytes (PBL) were subjected to FACS analysis by surface staining. Results were obtained from 11 patients with BDA and same patients as BDI.

## Slide 4
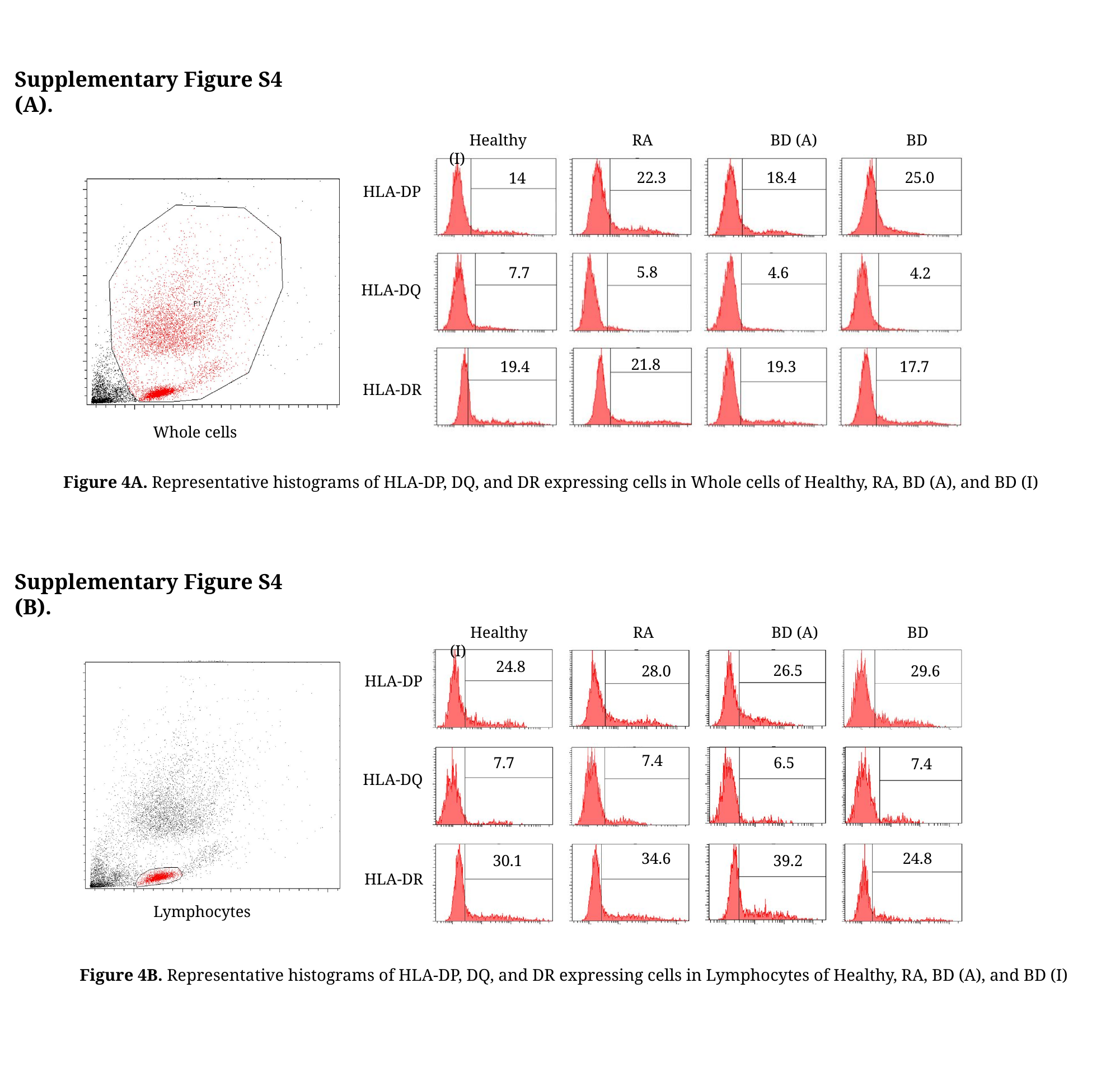

Supplementary Figure S4 (A).
 Healthy RA BD (A) BD (I)
18.4
25.0
22.3
14
HLA-DP
5.8
4.6
7.7
4.2
HLA-DQ
21.8
19.4
19.3
17.7
HLA-DR
Whole cells
Figure 4A. Representative histograms of HLA-DP, DQ, and DR expressing cells in Whole cells of Healthy, RA, BD (A), and BD (I)
Supplementary Figure S4 (B).
 Healthy RA BD (A) BD (I)
24.8
26.5
29.6
28.0
HLA-DP
7.4
7.7
6.5
7.4
HLA-DQ
24.8
34.6
39.2
30.1
HLA-DR
Lymphocytes
Figure 4B. Representative histograms of HLA-DP, DQ, and DR expressing cells in Lymphocytes of Healthy, RA, BD (A), and BD (I)

## Slide 5
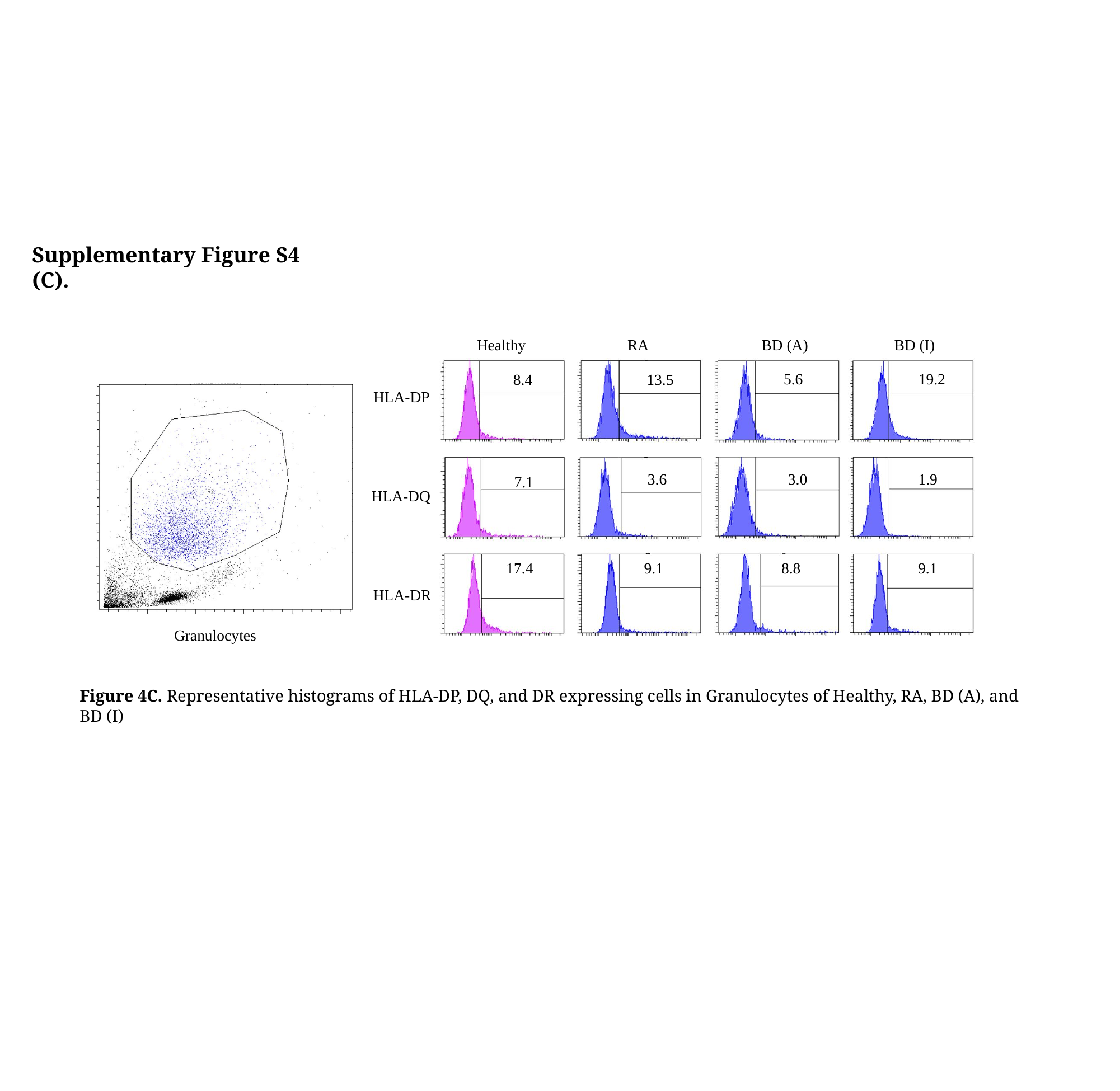

Supplementary Figure S4 (C).
 Healthy RA BD (A) BD (I)
5.6
19.2
13.5
8.4
HLA-DP
1.9
3.6
3.0
7.1
HLA-DQ
17.4
9.1
8.8
9.1
HLA-DR
Granulocytes
Figure 4C. Representative histograms of HLA-DP, DQ, and DR expressing cells in Granulocytes of Healthy, RA, BD (A), and BD (I)
