# Supplementary material for: Differences in Expression of Human Leukocyte Antigen Class II Subtypes and T Cell Subsets in Behçet’s Disease with Arthritis
Source: Int J Mol Sci. 2019 Oct 11;20(20):5044. doi: 10.3390/ijms20205044 (PMC6829274; doi:10.3390/ijms20205044)
Supplement: Supplementary file 1 [file ijms-20-05044-s001.zip › supplementary/Supplementary Tables.docx]

**Supplementary Table 1.**

**Table S1.** The frequencies of HLA subtype expressing cells in active Behçet’s Disease and inactive Behçet’s Disease patients

| Cell population | HLA subtype | BD Active (BDA, N=11) | BD Inactive (BDI, N=11) | P value |
| --- | --- | --- | --- | --- |
| Whole cells | HLA.DP | 16.4 (13.2 - 17.7) | 22.3 (14.2 - 32.8) |  |
|  | HLA.DQ | 4.4 (4.0 - 4.7) | 3.7 (2.8 - 5.9) |  |
|  | HLA.DR | 20.0 (15.5 - 21.7) | 13.9 (11.3 - 20.4) |  |
| Monocytes | HLA.DP | 85.2 (82.0 - 91.3) | 79.8 (76.4 - 84.7) |  |
|  | HLA.DQ | 36.4 (28.6 - 50.8) | 29.0 (21.8 - 49.4) |  |
|  | HLA.DR | 88.2 (85.1 - 92.5) | 86.3 (78.4 - 92.0) |  |
| Granulocytes | HLA.DP | 4.0 (3.2 - 6.6) | 18.9 (3.6 - 26.9) |  |
|  | HLA.DQ | 1.6 (1.1 - 2.1) | 0.9 (0.4 - 1.5) | 0.09 |
|  | HLA.DR | 6.7 (5.4 - 8.2) | 4.1 (3.2 - 5.3) | 0.05 |

**Supplementary Table 2.**

| **­­Number of Patients** | MTX | HCQ | Bucill  amine | SZP | Tacro  limus | Leflu  nomide | Biologics | Corticosteroid | NSAIDs |
| --- | --- | --- | --- | --- | --- | --- | --- | --- | --- |
| 1 | + | + | - | - | - | - | - | - | - |
| 2 | + | + | - | - | - | - | - | + | + |
| 3 | + | + | - | + | - | - | - | + | + |
| 4 | + | + | - | - | - | + | - | + | + |
| 5 | + | + | - | - | - | - | - | + | + |
| 6 | + | - | + | - | - | - | - | + | - |
| 7 | + | + | - | - | - | - | - | + | + |
| 8 | - | + | - | + | - | - | - | - | + |
| 9 | - | + | - | - | - | - | - | - | + |
| 10 | - | + | - | - | - | - | - | - | + |
| 11 | - | - | - | + | - | - | - | - | - |
| 12 | - | + | - | - | - | - | - | + | + |
| 13 | - | + | - | - | - | - | - | - | + |
| 14 | + | + | - | - | - | - | - | + | - |
| 15 | + | + | - | - | - | - | - | + | + |
| 16 | - | - | - | - | - | - | - | - | - |
| 17 | + | - | - | + | - | - | - | + | + |
| 18 | - | + | + | - | - | - | - | - | + |
| 19 | + | - | - | - | - | - | - | + | + |
| 20 | + | + | - | - | - | + | - | + | - |
| 2 1 | - | + | - | - | - | - | - | - | + |
| 22 | + | - | - | - | - | - | - | + | + |
| 2 3 | - | - | - | + | - | - | - | + | + |
| 24 | + | - | - | + | - | - | - | + | + |
| 25 | + | + | - | - | - | + | - | + | + |
| 26 | - | + | + | - | - | - | - | - | + |
| 27 | + | - | - | - | - | + | - | + | + |
| 28 | + | + | - | - | - | + | - | + | + |
| 29 | - | + | - | - | - | - | - | - | - |
| 30 | + | + | + | - | - | - | - | + | + |
| 31 | - | - | + | - | - | - | - | - | + |
| 32 | + | - | - | - | - | + | - | + | + |
| 33 | + | + | - | - | - | - | - | + | + |
| 34 | + | - | - | - | - |  | humira | + | + |
| 35 | + | + | - | + | - | - | - | + | + |
| 36 | + | + | - | - | - | - | - | + | + |

**Table S2:** Medication for patients with rheumatoid arthritis

**Note-** MTX: Methotrexate, HCQ: Hydroxychloroquine, SZP: Sulphasalazine
